# Paraneoplastic Pulmonary Embolism Revealing Metastatic Uterine Carcinosarcoma

**DOI:** 10.1155/crom/5340586

**Published:** 2026-02-27

**Authors:** Elias Tayar, Amira Bitar, Maroun Ibrahim

**Affiliations:** ^1^ Department of Internal Medicine, HCA Houston Healthcare Clear Lake, Webster, Texas, USA

## Abstract

**Background:**

Venous thromboembolism (VTE) can be a paraneoplastic phenomenon and may occasionally be the first manifestation of an occult malignancy. We present the case of an elderly woman whose “unprovoked” pulmonary embolism (PE) led to the diagnosis of metastatic uterine carcinosarcoma, an aggressive endometrial cancer subtype.

**Case:**

A 74‐year‐old postmenopausal woman with a history of uterine fibroids and prior breast ductal carcinoma in situ developed progressive dyspnea. She was found to have bilateral PEs with right heart strain and extensive bilateral deep vein thromboses (DVTs). Imaging also revealed ascites, widespread lymphadenopathy, and an 8‐cm uterine mass. Tumor marker evaluation showed a markedly elevated CA‐125 level. Diagnostic procedures including endometrial biopsy, ascitic fluid cytology, and lymph node biopsy confirmed Stage IVB uterine carcinosarcoma. She was managed with anticoagulation for VTE and initiated on systemic chemotherapy (carboplatin plus paclitaxel), given that surgical cure was not feasible due to metastases.

**Outcome:**

After treatment initiation, the patient′s respiratory status improved, and she was discharged on long‐term anticoagulation and chemotherapy, with plans for outpatient oncology follow‐up.

**Conclusion:**

This case highlights that an unexplained (unprovoked) PE in an older adult can be the harbinger of an underlying malignancy. In women, especially those with additional red flags such as abdominal distension or abnormal uterine bleeding, prompt evaluation for gynecologic cancers is warranted. Early identification of the occult cancer allowed appropriate therapy; however, uterine carcinosarcoma carries a poor prognosis once metastatic. Clinicians should maintain a high index of suspicion for hidden malignancy in cases of idiopathic VTE, as timely diagnosis can guide life‐saving interventions.

## 1. Introduction

Venous thromboembolism (VTE), including deep vein thrombosis (DVT) and pulmonary embolism (PE), is a classic manifestation of cancer‐associated hypercoagulability, often referred to as Trousseau′s syndrome. In 5%–10% of patients, an “unprovoked” VTE is the first clinical manifestation of an occult malignancy. Although mucinous gastrointestinal tumors are typically associated with paraneoplastic thrombosis, gynecologic cancers—particularly high‐grade endometrial cancers—also confer a substantial thrombotic risk. [1]

Uterine carcinosarcoma (malignant‐mixed Müllerian tumor) is a rare and aggressive subtype of endometrial carcinoma, accounting for < 5% of uterine cancers. [2] It is characterized by biphasic histology with malignant epithelial and mesenchymal components. Patients often present late with extrauterine disease, ascites, lymphadenopathy, and metastatic spread. Thromboembolism is a well‐recognized complication, especially in advanced stage disease. [2]

We report a case in which an unprovoked submassive PE was the sentinel event leading to the diagnosis of widely metastatic uterine carcinosarcoma. This case highlights the importance of considering occult malignancy in older adults presenting with idiopathic VTE, especially in the context of gynecologic symptoms or abdominal findings.

## 2. Case Presentation

A 74‐year‐old woman arrived in the emergency department with 1 week of gradually worsening shortness of breath and intermittent chest pressure. Over the preceding 2 weeks, she had also noticed increasing abdominal fullness and early satiety. She denied fever, cough, hemoptysis, or leg pain. When asked about gynecologic symptoms, she reported that she had experienced episodes of postmenopausal vaginal bleeding for several years. One year earlier, she had undergone imaging that showed a markedly thickened endometrium, and an endometrial biopsy had been recommended, but she never pursued the evaluation. Her past medical history included ductal carcinoma in situ of the breast treated with mastectomy 4 months before, without adjuvant therapy. She also had hypertension, diabetes, and hyperlipidemia.

On examination, she appeared mildly dyspneic but was not hypotensive. Her oxygen saturation was 97% on low‐flow oxygen. She had sinus tachycardia, clear breath sounds, and no peripheral edema. Her abdomen was visibly distended, and fluid could be appreciated on physical exam. A firm left inguinal lymph node measuring several centimeters was clearly palpable. There were no clinical signs of DVT in the extremities.

Initial laboratory studies revealed a microcytic iron‐deficiency anemia with a hemoglobin of 8.4 g/dL, accompanied by leukocytosis and a mildly elevated troponin. D‐dimer was positive. Because of her respiratory symptoms and elevated biomarkers, a CT pulmonary angiogram was performed. The scan revealed multiple bilateral pulmonary emboli and clear radiographic signs of right ventricular strain. However, the CT images also captured unexpected and concerning findings unrelated to the vascular clot: there were bulky lymph nodes throughout the abdomen and pelvis, an enlarged and heterogeneous uterine mass approximately 8 cm in size, moderate ascites, and several small pulmonary nodules that raised suspicion for metastatic disease.

Given these findings, a bilateral lower‐extremity Doppler ultrasound was obtained and confirmed extensive thromboses in both legs. The combination of bilateral DVTs and bilateral PEs strongly suggested a systemic prothrombotic state rather than a localized clot from a single limb.

The patient was admitted to the intensive care unit and started on intravenous heparin. She remained hemodynamically stable. An echocardiogram confirmed a moderate right ventricular dilatation. At this point, the clinical team strongly suspected an underlying malignancy and initiated a comprehensive oncologic workup.

Tumor markers were drawn shortly after admission. CA‐125 was markedly elevated, consistent with a gynecologic origin. Markers associated with gastrointestinal malignancy were normal. Gynecology was consulted, and a transvaginal ultrasound confirmed a thickened endometrium and an enlarged uterus. An endometrial biopsy was performed at the bedside. Interventional radiology obtained a biopsy of the enlarged inguinal lymph node, and a therapeutic paracentesis drained over 4 L of ascitic fluid, which was sent for cytology.

**Figure 1:**
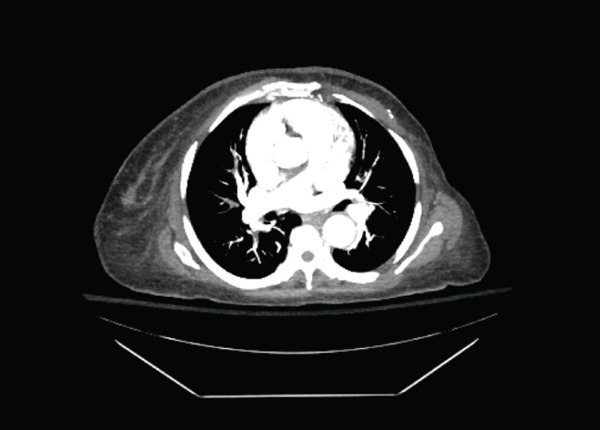
CTA confirming bilateral PE.

During the following days, her respiratory status improved on anticoagulation. Cytologic examination of the ascitic fluid returned positive for malignant cells consistent with Müllerian origin. Shortly afterward, pathology from the endometrial biopsy confirmed a high‐grade uterine carcinosarcoma. The lymph‐node biopsy also demonstrated metastatic carcinoma compatible with uterine primary. These findings established a diagnosis of FIGO Stage IVB uterine carcinosarcoma with metastases to lymph nodes, peritoneum, and likely the lungs. Her presentation with bilateral pulmonary emboli (Figure 1) was attributed to paraneoplastic hypercoagulability driven by her advanced cancer.

Once the diagnosis was confirmed, a multidisciplinary discussion was held involving oncology, gynecologic oncology, cardiology, and palliative care. Because of the extent of metastatic disease, surgical intervention such as hysterectomy was not considered beneficial. The plan focused on systemic therapy and continued anticoagulation. She was transitioned from heparin infusion to long–term low‐molecular‐weight heparin. For oncologic management, she began combination chemotherapy with carboplatin and paclitaxel, the standard first‐line regimen for advanced uterine carcinosarcoma. Tissue samples were sent for molecular profiling, including mismatch repair (MMR) status and HER2 testing, to determine whether immunotherapy or targeted therapy could play a role in future treatment.

Symptom management was an essential part of her care. Palliative care was involved early to assist with abdominal discomfort related to malignant ascites, fatigue, and general supportive needs. She tolerated her first cycle of chemotherapy well and gradually regained some functional strength with physical therapy. After stabilizing clinically, she was discharged home on therapeutic anticoagulation and scheduled for a close outpatient oncology follow‐up.

## 3. Discussion

This case illustrates a striking example of cancer‐associated thrombosis (Trousseau′s syndrome) revealing a hidden malignancy. An older patient with no obvious provoking factors for VTE was found to have bilateral pulmonary emboli and DVTs, which prompted a search for an underlying cause. The subsequent discovery of metastatic uterine carcinosarcoma underscores that “unprovoked” thromboembolism in an elderly patient should raise suspicion for occult cancer [3]. In fact, large prospective studies have demonstrated that approximately one in eight to 10 patients presenting with unprovoked VTE will be diagnosed with an underlying malignancy within 1 year [3]. In clinical practice, it is crucial to look beyond treating the thrombotic event itself and to remain vigilant for underlying systemic pathology. In our patient, several concomitant features, including malignant ascites, extensive lymphadenopathy, severe iron‐deficiency anemia, and a prolonged history of postmenopausal bleeding, strongly suggested an underlying malignant process. These red flags prompted an early and comprehensive diagnostic evaluation, ultimately leading to the diagnosis of advanced uterine carcinosarcoma. This case highlights the importance of integrating clinical, laboratory, and radiologic findings when evaluating patients with apparently unprovoked VTE. These prompted a comprehensive evaluation that confirmed an advanced gynecologic cancer. Had attention focused only on managing the PE without investigating further, the diagnosis of the uterine tumor might have been missed or delayed even longer.

The association between endometrial malignancies and VTE is well‐documented [4, 5]. Uterine carcinosarcoma, although rare, is an especially aggressive form of endometrial carcinoma and tends to metastasize early beyond the uterus [6, 7]. It also appears to confer a hypercoagulable state: case reports and series (including an autopsy case of fatal PE in carcinosarcoma) have drawn attention to the risk of thromboembolic complications in this cancer [4]. In a gynecologic oncology context, the presence of VTE often correlates with advanced tumor burden and aggressive histology [5]. For example, one predictive model study of endometrial cancer patients identified advanced stage (III/IV), high tumor grade, thrombocytosis, and elevated CA‐125 as significant risk factors for developing VTE [5]. Our patient had several of these risk features (high CA‐125, advanced Stage IV disease, and bulky tumor spread). The development of bilateral PEs in her case can, therefore, be seen as a manifestation of the malignancy′s aggressive nature. Furthermore, cancer‐associated thrombosis is not merely a coincidental finding but can portend a worse prognosis. It signals a biologically active tumor interacting with the coagulation system. Indeed, patients with malignancy who experience thromboembolism often have higher mortality rates than those who do not, likely reflecting the high tumor burden and virulence underlying the clotting event.

Uterine carcinosarcoma poses significant therapeutic challenges [6, 7]. By the time it presents (often with nonspecific symptoms or, as in this case, paraneoplastic phenomena), it is frequently at an advanced stage. A reported 5‐year overall survival is dismal in metastatic cases—on the order of 10%–20% [7]. Even with aggressive treatment, cures are rare once distant spread has occurred [7]. Management typically involves a combination of surgery, chemotherapy, and sometimes radiation for local control. In an early‐stage disease, comprehensive surgical staging and tumor debulking (hysterectomy with bilateral salpingo‐oophorectomy and lymphadenectomy) are performed [8]. However, in Stage IV or widely metastatic disease, surgery is usually palliative or omitted in favor of systemic therapy. The standard first‐line chemotherapy for carcinosarcoma is a platinum/taxane regimen (usually carboplatin plus paclitaxel), which has been shown to achieve better response rates and tolerability compared with older regimens (such as ifosfamide‐based combinations) [6, 7]. Our patient was started on this regimen, and initial tolerance was good. Response rates to this combination are on the order of 50%–60%, offering some patients a temporary remission or disease stabilization.

Given the aggressive behavior of carcinosarcomas, there has been interest in newer systemic therapies. Immunotherapy and targeted therapies are emerging options in advanced endometrial cancers. Current oncology guidelines recommend evaluating tumors for MMR deficiency or microsatellite instability, as tumors with MMR deficiencies can be treated with immune checkpoint inhibitors (like pembrolizumab or dostarlimab), which have shown considerable efficacy [8]. Additionally, even MMR‐proficient endometrial cancers that progress after first‐line chemotherapy may benefit from combination therapy with a checkpoint inhibitor plus targeted agents. For instance, the combination of pembrolizumab (PD‐1 inhibitor) and lenvatinib (an oral multikinase inhibitor) has become an approved second‐line treatment for advanced endometrial carcinomas that are not MMR‐deficient [8]. This regimen has achieved meaningful response rates in clinical trials, extending options for patients with otherwise refractory disease. In uterine carcinosarcoma, these approaches are being explored, given that carcinosarcoma is treated clinically as a high‐risk endometrial carcinoma [7, 8]. Our patient′s care team incorporated these considerations by sending her tumor for molecular profiling. Although such advanced therapies may not be curative in a Stage IV carcinosarcoma, they can potentially prolong survival and improve quality of life when standard chemotherapy alone is insufficient.

It is also worth noting the need to manage the cancer‐associated thrombosis alongside treating the cancer. Patients with active malignancy and VTE require long‐term anticoagulation, often for an indefinite duration while the cancer is present, due to the persistent hypercoagulable stimulus [5]. Low–molecular‐weight heparin has traditionally been the treatment of choice, though direct oral anticoagulants (DOACs) have also been shown effective in cancer‐associated VTE in recent trials [5]. In our case, anticoagulation was carefully managed given the concurrent risk of tumor‐related bleeding. A balance must be struck between preventing life‐threatening clots and avoiding hemorrhagic complications, especially when tumors (like an invasive uterine tumor) might bleed. Multidisciplinary collaboration—involving internists, oncologists, cardiologists, hematologists, and surgeons—is vital to navigate these complexities. In this patient′s course, close coordination among the specialties enabled prompt diagnosis and appropriate interventions (anticoagulation, biopsies, and initiation of chemotherapy) without undue delay.

Similar cases in the literature reinforce the key lessons from this report [3, 9]. For example, there are reports of patients with recurrent unexplained thromboses eventually found to have underlying endometrial cancer (presenting as Trousseau′s syndrome of malignancy) [9]. In another reported case, what was presumed to be an acute PE in a woman was later discovered to be tumor emboli in the pulmonary arteries from metastatic endometrial carcinoma—a dramatic reminder that malignancy can masquerade as a thrombotic disease in unusual ways [3].

Although occult malignancy is a well‐established risk factor for unprovoked DVT and PE, uterine carcinosarcoma remains a rare underlying etiology. Due to its aggressive nature and tendency for early dissemination, diagnosis is often delayed until advanced stages. Our case illustrates how paraneoplastic thrombosis may serve as an early clinical clue to this otherwise elusive malignancy. Early recognition of atypical features and prompt multidisciplinary evaluation are essential to facilitate timely diagnosis and initiation of appropriate oncologic management.

Our case is distinctive in that a paraneoplastic PE was the initial clue that, along with other clinical findings (ascites and unexplained bleeding), led to the timely diagnosis of a uterine carcinosarcoma, a relatively rare tumor. It underlines a critical teaching point: in any older adult with idiopathic VTE, clinicians should maintain a broad differential and consider occult malignancy. Moreover, if there are gynecologic signs such as abdominal distension or postmenopausal bleeding, one should have a high suspicion for uterine or ovarian cancers as potential culprits [6, 7].

## 4. Conclusion

This case report highlights how a “mysterious” unprovoked PE ultimately unmasked an advanced uterine carcinosarcoma in a 74‐year‐old woman. Her initial presentation with bilateral PEs, extensive bilateral DVTs, ascites, anemia, and lymphadenopathy exemplified an occult malignancy syndrome that could easily have been missed if attention was limited to treating the clot alone. Instead, recognizing the possibility of an underlying cancer prompted a thorough investigation that confirmed a metastatic uterine carcinosarcoma. Although her prognosis remains poor due to the extent of disease, identifying the cancer allowed for appropriate oncologic therapy and palliative care to begin. This case reinforces the adage that “cancer may clot before it is caught.” In clinical practice, the development of unprovoked VTE in a patient with other unexplained findings should prompt a diligent search for a hidden malignancy. Early detection of the cancer in such scenarios can be life‐saving or at least life‐prolonging, as it opens the door to targeted treatment. For women, in particular, never ignore postmenopausal bleeding or assume benign causes without adequate evaluation—a timely workup might diagnose a uterine tumor before it reaches an advanced, widely metastatic stage. Ultimately, maintaining a high index of suspicion and a multidisciplinary approach in cases of idiopathic thrombosis can significantly improve patient outcomes by unmasking treatable cancers that would otherwise remain undiagnosed.

## Funding

No funding was received for this manuscript.

## Disclosure

This research was supported (in whole or in part) by HCA Healthcare and/or an HCA Healthcare‐affiliated entity. The views expressed in this publication represent those of the authors and do not necessarily represent the official views of HCA Healthcare or any of its affiliated entities.

## Consent

Patient consent was obtained.

## Conflicts of Interest

The authors declare no conflicts of interest.

## Data Availability

The data that support the findings of this study are available on request from the corresponding author. The data are not publicly available due to privacy or ethical restrictions.
